# Shouting out loud: signaling modules in the regulation of stomatal development

**DOI:** 10.1093/plphys/kiaa061

**Published:** 2020-12-18

**Authors:** Arvid Herrmann, Keiko U Torii

**Affiliations:** Howard Hughes Medical Institute and Department of Molecular Biosciences, University of Texas at Austin, Austin, Texas 78712, USA

## Abstract

Stomata are small pores on the surface of land plants that facilitate gas exchange for photosynthesis while minimizing water loss. The function of stomata is pivotal for plant growth and survival. Intensive research on the model plant Arabidopsis (*Arabidopsis thaliana*) has discovered key peptide signaling pathways, transcription factors, and polarity components that together drive proper stomatal development and patterning. In this review, we focus on recent findings that have revealed co-option of peptide-receptor kinase signaling modules—utilized for diverse developmental processes and immune response. We further discuss an emerging connection between extrinsic signaling and intrinsic polarity modules. These findings have further enlightened our understanding of this fascinating developmental process.

## Teaser

Co-opted peptide-receptor kinase signaling modules and intrinsic polarity cues regulate stomatal development.

## Introduction

**Figure 1:**
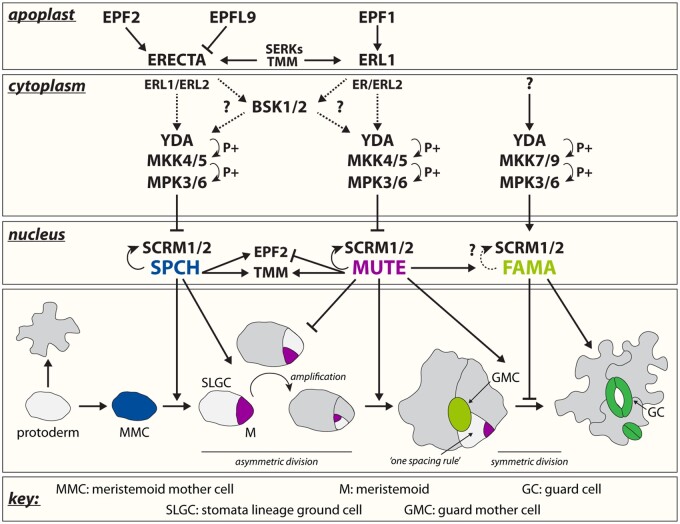
Peptide–receptor signaling throughout stomata development. Overview of the different receptor peptide signaling modules and their main downstream components regulating cell-state transitional steps within the stomatal cell lineage. Members of the EPF family, EPF1, EPF2, and EPFL9/STOMAGEN, are secreted from stomatal precursors and underlying mesophyll cells into the apoplast and competitively bind to three cell surface LRR–RLKs, ERECTA (ER), ER-LIKE 1 (ERL1), and ERL2. Together with the LRR receptor protein, TMM and BRI1-ASSOCIATED RECEPTOR KINASE/SOMATIC EMBRYOGENESIS RECEPTOR KINASE (BAK/SERK) family RLKs, they form a ternary receptor complex. This complex activates the downstream MAPK cascade (arrow, P+) that is composed of YODA (YDA) MAPKKK, two redundant MAPKKs (MKK4 and MKK5) and two, redundant MAPKs (MPK3 and MPK6) possibly through two functional redundant members of the BSK family (question marks; cytoplasm). The activated MAPK cascade in return controls the consecutive activities of three bHLH TFs and their partner bHLH proteins SCREAM (SCRM) and SCRM2 (nucleus). While SPEECHLESS (SPCH) and MUTE are negatively regulated through TF inhibition by the MAPK cascade, FAMA is activated by the latter to inhibit further symmetric cell division and promote cell differentiation. Each TF pair is specifically expressed during the timely progression of stomata development (color indicated). In the model plant Arabidopsis, stomata development occurs by the initiation of an asymmetric division of protodermal cells called MMCs (blue). The ACD generates a small meristemoid (purple) and a large SLGC (light grey). The meristemoid can undergo one to three rounds of asymmetric divisions in an inward-spiral manner to produce additional amplicons surrounded by SLGCs. Stomata development ends when the late meristemoid differentiates into a GMC (light green), which then will further divide symmetrically to generate a pair of GCs (dark green).

Stomata are turgor-driven microscopic valves in the epidermis of aerial regions of land plants. The controlled opening and closing of the stomatal pores is essential to the regulation of gas exchange and water loss by the plant (Chaerle et al., 2005). In addition, maintaining proper stomatal density, distribution, and development are pivotal for plant survival. In Arabidopsis, a dicot plant, stomatal development occurs through the initiation of an entry division of a subset of undifferentiated protodermal cells called meristemoid mother cells (MMCs). The asymmetric cell division (ACD) of the MMC generates a small, triangular-shaped meristemoid (Ms), and its sister cell, a stomatal lineage ground cell (SLGC). The Ms possess a stem cell-like character and can undergo rounds of ACDs in an inward spiralling manner. Late Ms lose their stem cell-like potential and differentiate into a round guard mother cell (GMC). Finally, the GMC will further divide symmetrically to generate a pair of guard cells (GCs) that surround a pore (Nadeau and Sack, 2002; Bergmann and Sack, 2007; Pillitteri and Torii, 2012). To optimize stomatal distribution during leaf expansion, additional spacing divisions occur away from the existing stomata. This process follows in accordance with the “one-cell spacing rule,” in which stomata should not directly contact each other. Consequently, young SLGCs are allowed to re-establish MMC identity, thus undergoing asymmetric spacing division to form secondary stomata (Nadeau and Sack, 2002). The rest of the protodermal cells, which are not destined to become stomata, differentiate into pavement cells, which make up the rest of the epidermis, acquiring the characteristic puzzle-like pattern (Figure 1).

The genetic control of stomatal development requires the consecutive activity of three basic helix-loop-helix (bHLH) transcription factors (TFs), SPEECHLESS (SPCH), MUTE, and FAMA, which drive stomatal initiation, proliferation, and differentiation, respectively (Figure 1; Ohashi-Ito and Bergmann, 2006; MacAlister et al., 2007; Pillitteri et al., 2007). These TFs act in coordination with two additional, redundant bHLH TFs—ICE1/SCREAM (SCRM) and SCRM2—which are expressed throughout the stomatal lineage, forming heterodimer TF complexes (Kanaoka et al., 2008; Pillitteri and Torii, 2012). Receptor-like kinases (RLKs) coupled with a mitogen‐activated protein kinase (MAPK) signaling cascade, mediated by YODA (YDA, MAPKKK)—MKK4/5 (MAPKKs)—MPK3/6 (MAPKs), negatively regulate these core bHLH heterodimers to enforce proper stomatal patterns (Müller et al. 2010; Meng et al., 2012). In return, bHLH heterodimers induce expression of some of the key signaling components, thereby forming a negative-feedback loop (Lau et al., 2014; Horst et al., 2015; Qi and Torii, 2018). This review aims to summarize the complex signaling pathways that orchestrate stomatal development, synthesize how peptide ligands and receptors are co-opted for different developmental programs, and explore an emerging connection between extrinsic signaling and intrinsic polarity modules in the process (Figure 1). Please see Table 1 for quick guides of key regulators of stomatal development discussed in this review article.

**Advances**

- Since the discovery of ERf and TMM as a co-receptor controlling stomatal development, various upstream and downstream components have been identified, aiding in the formation of specific peptide–receptor signaling modules and signal transduction.
- These peptide–receptor signaling modules can act in both paracrine and autocrine manners, dependent on the developmental stage of the respective stomatal cell.
- The peptide–receptor signaling modules have been co-opted to regulate arrays of developmental programs and intersect with immunity and environmental response.
- The interplay of extrinsic signals and intrinsic polarity cues, that provide spatial information during cell division, are still not fully understood. However, the discovery of novel polarity proteins and regulatory mechanisms are now unravelling a molecular intersection of different protein modules during stomatal development.

**Table 1:** Regulators of stomatal development

Component	Gene	AGI	Reference (in the context relevant to this review)
Peptides	*EPF1*	At2g20875	Hara et al., 2007, 2009; Dong et al., 2009; Ohki et al., 2011; Lee et al., 2012; Qi et al., 2017
	*EPF2*	At1g34245	MacAlister et al., 2007; Hara et al., 2009; Ohki et al., 2011; Lee et al., 2012; Lau et al., 2014; Horst et al., 2015; Lin et al., 2017
	*EPFL9/STOMAGEN*	At4g12970	Hunt and Gray, 2009; Kondo et al., 2009, 2010; Hunt et al., 2010; Sugano et al., 2010; Ohki et al., 2011; Lee et al., 2012, 2015
	*EPFL2*	At4g37810	Tameshige et al., 2016; Kawamoto et al. 2020
	*EPFL4/CLL2*	At4g14723	Abrash and Bergmann, 2010; Abrash et al., 2011; Lin et al., 2017
	*EPFL6/CHAL*	At2g30370	Abrash and Bergmann, 2010; Abrash et al., 2011; Lin et al., 2017
	*CLE9*	At1g26600	Qian et al., 2018; Vatén et al., 2018
	*CLE10*	At1g69320	Qian et al., 2018; Vatén et al., 2018
Receptor	*ER*	At2g26330	Rédei, 1992; Torii et al., 1996; Shpak et al., 2003, 2004; Woodward et al., 2005; Uchida et al., 2012; Bemis et al., 2013; Chen et al., 2013; Ikematsu et al., 2017; Kosentka et al., 2017; Lin et al., 2017; Hohmann et al., 2020
	*ERL1*	At5g62230	Torii et al., 1996; Shpak et al., 2003, 2004; Uchida et al., 2012; Bemis et al., 2013; Chen et al., 2013; Ikematsu et al., 2017; Qi et al., 2020, 2017
	*ERL2*	At5g07180	Torii et al., 1996; Shpak et al., 2004, 2003; Uchida et al., 2012; Bemis et al., 2013; Chen et al., 2013; Ikematsu et al., 2017
	*HSL1*	At1g28440	Qian et al., 2018
	*FLS2*	At5g46330	Hohmann et al., 2020
Co-receptor	*TMM*	At1g80080	Yang and Sack, 1995; Geisler et al., 1998; Nadeau and Sack, 2002; Bhave et al., 2009; Dong et al., 2009; Lin et al., 2017
	*SERK1*	At1g71830	Hecht et al., 2001; Wang et al., 2008, 2015; Meng et al., 2015
	*SERK2*	At1g34210	Hecht et al., 2001; Wang et al., 2008, 2015; Meng et al., 2015
	*SERK3/BAK1*	At4g33430	Hecht et al., 2001; Chinchilla et al., 2007; Heese et al., 2007; Wang et al., 2008; Postel et al., 2010; Roux et al., 2011; Santiago et al., 2013; Sun et al., 2013a, 2013b; Aan den Toorn et al., 2015; Meng et al., 2015; Hohmann et al., 2018; Perraki et al., 2018; Qian et al., 2018
	*SERK4*	At2g13790	Hecht et al., 2001; Wang et al., 2008; Postel et al., 2010; Meng et al., 2015
Downstream components	*BSK1*	At4g35230	Kim and Wang, 2010; Shi et al., 2013; Neu et al., 2019; Nolan et al., 2020; Wang et al., 2020
	*BSK2*	At5g46570	Kim and Wang, 2010; Shi et al., 2013; Neu et al., 2019; Nolan et al., 2020; Wang et al., 2020
	*BIK1*	At2g39660	Lozano-Durán and Zipfel, 2015; Chen et al., 2019
	*YDA*	At1g63700	Bergmann et al., 2004; Lukowitz et al., 2004; Meng et al., 2012
	*MKK4*	At1g51660	Wang et al., 2007; Meng et al., 2012
	*MKK5*	At3g21220	Wang et al., 2007; Meng et al., 2012
	*MKK7*	At1g18350	Lampard et al., 2009; Meng et al., 2012
	*MKK9*	At1g73500	Lampard et al., 2009; Meng et al., 2012
	*MPK3*	At1g45640	Wang et al., 2007; Lampard et al., 2008, 2009; Zhang et al., 2015, 2016; Putarjunan et al., 2019
	*MPK6*	At2g43790	Wang et al., 2007; Lampard et al., 2008, 2009; Zhang et al., 2015, 2016; Putarjunan et al., 2019
	*MKP1*	At3g55270	Tamnanloo et al., 2018
	*PP2A-A1*	At1g25490	Bian et al., 2020
	*PP2A-A2*	At3g25800	Bian et al., 2020
	*PP2A-A3*	At1g13320	Bian et al., 2020
Polarity components	*BASL*	At5g60880	Dong et al., 2009; Zhang et al., 2015, 2016
	*POLAR*	At4g31805	Pillitteri et al. 2011; Houbaert et al., 2018
	*BIN2*	At4g18710	Gudesblat et al., 2012; Khan et al., 2013; Houbaert et al., 2018
	*BRX*	At1g31880	Rowe et al., 2019
	*BRXL1*	At2g35600	Rowe et al., 2019
	*BRXL2*	At3g14000	Rowe et al., 2019
	*BRXL3*	At1g54180	Rowe et al., 2019
	*BRXL4*	At5g20540	Rowe et al., 2019
	*MASS1*	At1g80180	Xue et al., 2020
	*MASS2*	At1g15400	Xue et al., 2020
	*MASS3*	At5g20100	Xue et al., 2020
bHLH TFs	*SPCH*	At5g53210	Ohashi-Ito and Bergmann, 2006; MacAlister et al., 2007; Pillitteri et al., 2007; Lampard et al., 2008; Lau et al., 2014; Horst et al., 2015; Putarjunan et al., 2019
	*MUTE*	At3g06120	Ohashi-Ito and Bergmann, 2006, MacAlister et al., 2007; Pillitteri et al., 2007, Qi et al., 2017; Han et al., 2018
	*FAMA*	At3g24140	Ohashi-Ito and Bergmann, 2006; MacAlister et al., 2007; Pillitteri et al., 2007; Lampard et al., 2009
	*SCRM/ICE1*	At3g26744	Kanaoka et al., 2008; Putarjunan et al., 2019
	*SCRM2*	At1g12860	Kanaoka et al., 2008; Putarjunan et al., 2019
Cytokinin components	*ARR16*	At2g40670	Vatén et al., 2018
	*ARR17*	At3g56380	Vatén et al., 2018
Auxin transporters	*PIN1*	At1g73590	Chen et al., 2013; Le et al., 2014
	*PIN2*	At5g57090	Le et al., 2014
	*PIN3*	At1g70940	Le et al., 2014
	*PIN4*	At2g01420	Le et al., 2014
	*PIN7*	At1g23080	Le et al., 2014
Aux/IAAs	*IAA17/AXR3*	At1g04250	Balcerowicz et al., 2014
	*IAA12/BDL*	At1g51950	Zhang et al., 2014
Auxin response factor	*ARF5/MP*	At1g19850	Zhang et al., 2014

## RLK signaling complexes ensure stomata development inhibition

**Figure 2:**
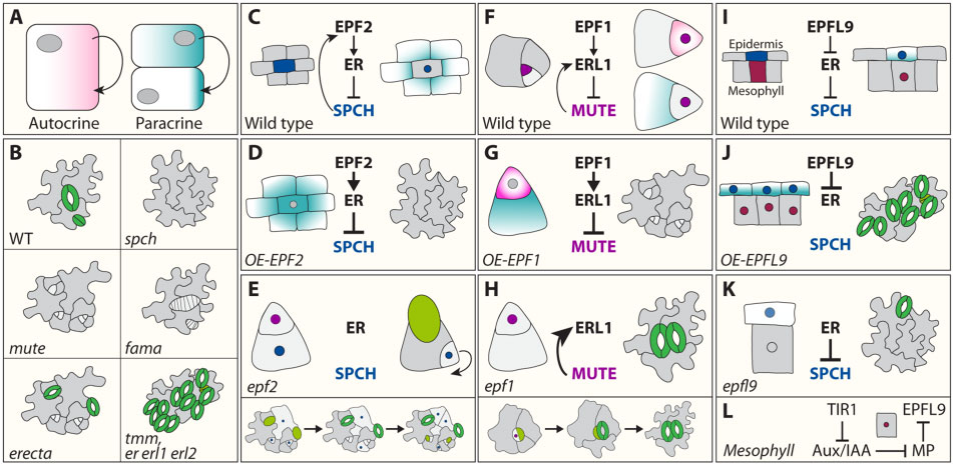
Overview of stomata mutant phenotypes and signaling modes of action during stomata development. (A), Different modes of action during cell signaling. During autocrine signaling, a cell secretes a messenger (arrow), which leads to changes in the same cell after binding to cell surface receptors (light red). Paracrine signaling from a cell induces a cellular response in a nearby cell (arrow) in a non-cell autonomous manner (light blue). (B), Mutant phenotypes of key stomatal components. While loss of function mutations in the bHLH TFs SPCH and MUTE lead to phenotypes only consisting of epidermal cells in case of *spch* and arrested meristemoids in case of *mute*, piled up symmetric divisions in GMCs can be observed for *fama* mutants. This indicates that each individual TF is required for a specific cell differentiation step during stomata development ontogenesis. Although an increased number of ACDs in the *er* single mutant can be observed, only the *erf* triple mutant (*er erl1 erl2*) or loss of *tmm* exhibit severe stomatal clustering. Uncoupling the functional redundancy of the ERf members identified specific peptide–receptor pairs, which contribute to different steps during stomata development (C–H). SPCH-induced EPF2-dependent paracrine signaling by MMCs (light blue) is perceived by ER in neighboring protodermal cells to inhibit SPCH (dark blue) activity (C). Modulation in EPF2 activity can cause an epidermis devoid of stomata when overexpressed (D) or piled up asymmetric division in *epf2* mutants (E) since SPCH activity is not downregulated by the EPF–ER signaling module. MUTE (purple) activity depends on both EPF1-dependent autocrine (light blue) and paracrine (light red) signaling. MUTE directly induces ERL1 expression, while at the same time ERL1 perceives EPF1 signal in SLGCs (paracrine) as well meristemoids (autocrine) to inhibit MUTE activity (F). This negative feedback loop ensures the right amount of MUTE activity provided by the EPF1–ERL1 signaling module for proper GC differentiation. While overexpression of EPF1 causes meristemoid arrest due to excessive activation of the ERL1-driven signaling pathway (G), loss of *EPF1* results in stomata clustering due to excessive MUTE activity (H). I, EPFL9, also known as STOMAGEN, is expressed in the mesophyll (dark red) and positively regulates stomatal development through a competitive mode of action with EPF1 and EPF2 for receptor binding on ERf members. While overexpression or exogenous application of EPFL9/STOMAGEN induces paracrine signaling, leading to numerous clustered stomata (J), *epfl9/stomagen* confers reduced stomatal density (K). Intriguingly, the molecular control of *EPFL9/STOMAGEN* expression within the mesophyll is tied to auxin signaling. Repression of EPFL9/STOMAGEN is ensured by ARF5/MONOPTEROS (MP), which in return is under the control of specific AUX/IAA BODENLOS (L).

Plants possess a battery of RLKs. Those RLKs with an extracellular leucine-rich repeat (LRR) domain play key roles in numerous aspects of plant development and immune response (Albert et al., 2020; Gou and Li, 2020). Stomatal development is not an exception. Three members of the ERECTA-family (ERf) LRR–RLK, namely ERECTA (ER), ERECTA-LIKE1 (ERL1), and ERL2, regulate plant organ growth, inflorescence elongation, vascular patterning, and leaf shape (Figure 1; Torii et al., 1996; Shpak et al., 2003, 2004; Uchida et al., 2012; Bemis et al., 2013; Chen et al., 2013; Ikematsu et al., 2017). The same ERf is in addition crucial for stomata patterning and differentiation. *ER*, which is highly expressed in protodermal cells, primarily acts to repress asymmetric entry division of MMCs, reflected by the increased number of ACDs in the *er* single mutant. Meanwhile, both *ERL1* and *ERL2* subsequently function to prevent M differentiation. In the *erl1 erl2* double mutant, Ms prematurely differentiate into GMCs, underlining their important role in maintaining the amplifying potential of Ms (Shpak et al., 2005). Severe stomatal clustering is observed when all ERf members lose function (*er erl1 erl2*; Figure 2B;Shpak et al., 2005).

An LRR receptor-like protein (LRR–RLP), TOO MANY MOUTHS (TMM), lacks the characteristic intracellular kinase domain of LRR–RLKs (Yang and Sack, 1995; Bhave et al., 2009). *tmm* mutants show altered stomatal patterning reminiscent of *er erl1 erl2* triple mutants, i.e. clustered and numerous stomata in the epidermis of cotyledons and leaves (Figure 2B; Yang and Sack, 1995). However, in contrast to *er erl erl2*, hypocotyls and stems of *tmm* are devoid of stomata, indicative of an additional complex regulatory function of *TMM* in the formation of stomata (Geisler et al., 1998). Further detailed genetic analyses reveal a role for *TMM* activity in precursor of stomatal lineage cell fate and progression, in an organ- and region-specific manner (Bhave et al., 2009).

Taking the phenotypes of both *er-*family members and *tmm* into consideration, one might wonder about their genetic and molecular relationships. Initial genetic evidence has hinted that these proteins contribute in combination to determine stomatal-lineage cell fate, possibly through dimerization (Shpak et al., 2005). Indeed, recent structural and biochemical analyses show that ERf members are able to form constitutive heterodimer complexes with TMM, thereby pre-forming a ligand-binding pocket (Lin et al., 2017). Interestingly, depending on the specific ligands, the heterodimerization of ERf and TMM will be unfavored, and this ligand-based discrimination of receptor heterodimerization is likely the molecular basis of complex TMM function (Figures 1 and 2).

## Secreted EPF peptides form different receptor-peptide signaling modules to regulate stomatal cell fate

A central question, then, is identifying ligands perceived by ERf and TMM. A family of secreted cysteine-rich peptides, EPIDERMAL PATTERNING FACTOR (EPF)/EPF-LIKE (EPFL), has been identified as a regulator of stomatal development (Hara et al., 2007, 2009; Hunt and Gray, 2009; Kondo et al., 2009; Hunt et al., 2010; Sugano et al., 2010; Lee et al., 2015; Lin et al., 2017). The predicted mature EPF/EPFL peptides possess six or eight cysteines that form disulfide bonds, constructing a loop region and a functionally crucial scaffold. Intriguingly, structural analyses by nuclear magnetic resonance combined with loop region swapping experiments reveal that the variable loop region determines the antagonistic activity of EPF2 with EPFL9/STOMAGEN (Ohki et al., 2011). EPF1 and EPF2 are both secreted from stomatal precursor cells and produce an inhibitory effect on stomatal lineage formation, while mesophyll cell-derived EPFL9/STOMAGEN peptides promote stomatal lineage proliferation through a competitive binding on selected ERf members (Hunt et al., 2010; Lee et al., 2015). Although EPF1 and EPF2 both negatively regulate stomatal development, careful genetic analyses have uncovered a more distinctive role for each signaling peptide (Hara et al., 2009; Hunt and Gray, 2009).

Genetic studies have identified EPF2 as an inhibitor of protodermal cells entering the stomatal lineage. Consistently, *EPF2* is expressed in early stomatal lineage precursors, MMCs, and early Ms (Hara et al., 2009). In early developmental stages, the *epf2* mutant displays numerous small, undifferentiated epidermal cells, resulting in more GCs and smaller pavement cells upon their differentiation. This phenotype is similar to those observed in a dominant negative mutant form of ER or *SPCH* overexpression, in which uninhibited asymmetric entry divisions occur during early stages of the stomatal lineage (Figure 2; Shpak et al., 2003; MacAlister et al., 2007; Pillitteri et al., 2007; Hara et al., 2009; Lee et al., 2012). Conversely, ectopic expression of *EPF2* or application of a bioactive EPF2 peptide results in the development of an epidermis composed of only pavement cells, identical to the *spch* mutant (Figure 2, B and D; Lee et al., 2012). Combined with the opposing *epf2* and *spch* phenotypes (Figure 2, B and E) and SPCH ChIP-seq assays reporting that *EPF2* is a direct SPCH target (Lau et al., 2014), these data support a model in which EPF2 is secreted from *SPCH*-expressing MMCs to neighboring cells and acts as a cell-to-cell (paracrine) signal downregulating SPCH activity. This negative feedback loop prevents surrounding protodermal cells from entering the stomatal lineage (Figure 2C; Lau et al., 2014; Horst et al., 2015). Experimental evidence has elucidated how this paracrine signal is perceived by neighboring cells. Exogenous EPF2 application on plants expressing the dominant negative form of ER alleviates the effects of the latter, indicating that EPF2 and ER act in the same pathway. Consistently, biochemical and structural data have identified the pre-formed, heterodimeric complex of ER and TMM as a binding partner of EPF2 (Lee et al., 2015; Lin et al., 2017). Altogether, this heterodimeric receptor-peptide signaling complex transduces the signal further into the cell (discussed below) to restrict entry into the stomata lineage through SPCH inhibition (Figure 2, C–E).

While EPF2 functions during early stomatal development, *EPF1* is expressed in later stages, including in late Ms, GMCs, and young GCs, and serves as a positional cue providing spatial information to maintain the “one-cell spacing rule” between developing stomata (Figure 2, F–H; Hara et al., 2007; Lee et al., 2012). Initially identified in a large genome-wide screen for secreted peptides, the *epf1* single mutant exhibits stomatal pairwise clustering, similar to the phenotype of plants expressing a dominant negative mutant version of *ERL1* (Figure 2H; Hara et al., 2007; Lee et al., 2012). Conversely, overexpression of *EPF1*, or exogenous EPF1 application, does not affect MMC formation but results in an epidermis composed of arrested Ms, resembling the loss-of-function mutation in *MUTE* (Figure 2G; Pillitteri et al., 2007; Lee et al., 2012).

Initially, it was proposed that paracrine signaling from differentiating Ms to neighboring SLGCs must occur to preserve the orientation of secondary asymmetric spacing division, thus maintaining the “one spacing” rule. Indeed, studies localizing the known polarized plasma membrane marker BREAKING OF ASSYMMETRY IN THE STOMATAL LINEAGE (BASL), which predicts the future division plane at the opposing end of the future M, showed that its distribution is flawed in both *tmm* and *epf1* mutants (Dong et al., 2009). Incorrect positioning of BASL crescent accumulation in *tmm* and *epf1* SLGC instructs incorrect asymmetric spacing division orientation. However, the overall accumulation distal to the new M in both *tmm* and *epf1* single mutants is still present (Dong et al., 2009). Since the double mutant combinations of *tmm basl* as well as *epf1 basl* exhibit an additive phenotype, BASL likely acts independently of both TMM and EPF1. How exactly the EPF1-ERL1 signaling pathway might influence the location of BASL polarization, and stomatal lineage cell polarity, is still not understood. Taken together, these findings challenge the notion of a paracrine signaling module from differentiating M to neighboring SLGCs.

In spite of this, a new model has been proposed which combines the traditional view of EPF/EPFL family peptides as non-cell autonomous signals with the additional contribution of EPF1 in autocrine regulation of GMC differentiation (Figure 2F; Qi et al., 2017). MUTE directly induces *ERL1* expression, while at the same time ERL1 perceives EPF1 signal to inhibit MUTE activity, creating a negative feedback-loop ensuring the presence of the right amount of MUTE for GC differentiation. This delicate balance between transcriptional regulation and signal transduction can prevent extra symmetric divisions of Ms and GMCs, and an impaired signal transduction by the *tmm* mutation results in stomatal pairwise clustering, independent of known polarity contributors (Qi et al., 2017). This is likely due to excessive MUTE activity, since *MUTE* overexpression could trigger numerous GMC symmetric divisions (Han et al., 2018). Moreover, this autocrine inhibition of stomata cell fate can lead to severe M arrest, possibly due to excessive activation of the ERL1-driven signaling pathway, a phenotype also observed in plants with overexpression or exogenous treatment of *EPF1* (Lee et al., 2012; Qi et al., 2017). This model is also supported by the finding that absolute co-expression of ERL1 and MUTE, driven by the *MUTE* promoter in an *er*-family triple mutant background, results in the same severe phenotype. Taking the functional redundancy of the ERf members in consideration, the ERL1, ER, and ERL2 receptor populations expressed in SLGCs are likely buffering the extreme amount of EPF1 peptide ligands secreted from Ms in a paracrine manner to ensure an adequate autocrine inhibition for proper stomata development (Abrash et al., 2011; Uchida and Tasaka, 2013; Qi et al., 2017). Such receptor buffering systems are known in the context of plant development. A genetic study suggests that, during shoot apical meristem formation, BARELY ANY MERISTEM (BAM) receptors sequester peptide ligands at the flanks of the meristems. This paracrine impoundment establishes a buffer around the meristems which prevents these peptide ligands from disturbing the delicate balance necessary for stem cell maintenance (DeYoung and Clark, 2008).

In contrast to EPF1 and EPF2, EPFL9 (also known as STOMAGEN) positively regulates stomatal development (Kondo et al., 2009; Hunt et al., 2010; Sugano et al., 2010). Expressed in immature mesophyll cells, EPFL9/STOMAGEN competes for receptor binding with EPF1 and EPF2, eliminating the negative impact of both peptides on their own downstream signal transduction (Figure 2, I–L; Lee et al., 2012, 2015). While overexpression or exogenous application of EPFL9/STOMAGEN leads to numerous clustered stomata (Figure 2J), reduced *EPFL9/STOMAGEN* transcripts in RNAi lines results in reduced stomatal density (Figure 2K), suggesting that the elaborate balance between EPF1, EPF2, and EPFL9/STOMAGEN is necessary to further fine tune stomatal distribution (Kondo et al., 2009; Hunt et al., 2010; Sugano et al., 2010; Lee et al., 2012, 2015). The molecular control of *EPFL9/STOMAGEN* expression, however, underlies the AUXIN RESPONSE FACTOR5 (ARF5)/MONOPTEROS (MP), which directly binds to auxin-responsive elements within the promoter region of *EPFL9/STOMAGEN* to repress its expression (Figure 2L; Zhang et al., 2014). Consistently, loss of MP or stabilizing its suppressor AUX/IAA BODENLOS (BDL)/INDOLE-3-ACETIC ACID INDUCIBLE12 (IAA12), results in an increase of stomatal density due to elevated EPFL9/STOMAGEN levels. Taken together, these findings support the negative role of auxin in stomatal development through the inhibition of *EPFL9/STOMAGEN* expression within the mesophyll.

The competitive binding of these peptide ligands with different activities raises the question as to which mode ERf receptor kinase responds to the individual peptides at the subcellular level. A recent study combining genetic, pharmacological, and live cell imaging analyses revealed new insights into the initial subcellular behaviors of the receptor ERL1 upon ligand perception. EPF1, which activates the inhibitory stomatal signaling cascade, triggers TMM-dependent ERL1 internalization into the intraluminal vesicles of multi-vesicular bodies/late endosomes for subsequent vacuolar degradation (Qi et al., 2020). Conversely, upon EPFL9/STOMAGEN perception, ERL1 is retained at the endoplasmic reticulum, likely due to impaired ERL1 endocytosis (Qi et al., 2020). It is not clear, however, whether the retained endoplasmic reticulum accumulation of ERL1 originates from former ERL1–TMM receptor complexes recycled back to the endoplasmic reticulum and/or whether newly synthesized ERL1 receptor molecules are stalled to decrease the overall ERL1 receptor population at the cell surface. It is reasonable to predict that ERfs possess specific short-sequences within their cytoplasmic domains, which direct routing and trafficking decisions. Indeed, the dominant-negative ERL1, which lacks the entire cytoplasmic domain, is insensitive to either EPF1 or EPFL9/STOMAGEN application and predominantly remains at the plasma membrane (Qi et al., 2020). It would be interesting to examine whether particular adapter coat proteins recognize specific domains of ERfs and how post-translational modifications (PTMs), such as phosphorylation or ubiquitination, might affect peptide ligand perception or contribute to their molecular sorting. At present, functional evidence for PTMs is only inferred from site-directed mutagenesis studies of ER (Kosentka et al., 2017; Perraki et al., 2018). Identifying these mechanisms will help illuminate the molecular relationships between antagonistic peptides at the subcellular level.

## Check and balance—how CLE peptides influence stomatal-lineage division patterns

Despite the significant roles of secreted EPF/EPFL family members, it is not the only peptide family that influences stomatal patterning. In flowering plants, the CLAVATA3/ENDOSPERM SURROUNDING REGION-RELATED (CLE) peptide family of post-translationally modified dodecapeptides regulate a wide range of biological processes in stem cell homeostasis, as well as in response to phytohormone signaling (Cock and McCormick, 2001; Fletcher, 2020). The potential role of CLE peptides was first implicated from a transcriptome study: *CLE9* accumulated highly and specifically in *mute scrm-D* seedlings, in which the epidermis was solely composed of Ms (Pillitteri et al., 2011). More recent work has shown that CLE9 and CLE10 have dual functions in roots and in leaves (Qian et al., 2018). In the root meristem, CLE9/10 peptides form signaling complexes with BAM receptors to repress the periclinal cell division of xylem precursor cells. In leaves, CLE9/10 negatively regulates the division of MMCs (Qian et al., 2018). There, CLE9/10 peptides are perceived by the LRR receptor kinase HAESA-LIKE1 (HSL1), enabling the activation of the known components of the MAPK cascade, resulting in the phosphorylation and destabilization of SPCH (Qian et al., 2018). Since the exogenous application of CLE9/10 peptide in the *er erl1 erl2* triple mutant decreases the number of GCs, it seems likely that this receptor-peptide module functions independently of the known ERf-EPFs signaling pathway to modulate stomata density.

Another study suggests that CLE9/10 peptides act downstream of cytokinin signaling in a non-cell autonomous manner via SPCH (Vatén et al., 2018). In wild-type plants, both *CLE9* and the cytokinin signaling effector gene *ARABIDOPSIS RESPONSE REGULATOR16* (*ARR16)*, a M-expressed gene acting as negative regulator for the latter, are directly induced by SPCH in Ms (Ren et al., 2009; Pillitteri et al., 2011; Lau et al., 2014; Vatén et al., 2018). During amplifying divisions, the induction of CLE9/CLE10 peptides in Ms suppress further SLGC division potential, possibly upstream of ARR16/17. On the other hand, the sustained ARR16/ARR17 effect in SLGCs reduces their sensitivity to cytokinin and subsequently lowers the probability of undergoing a spacing division. Intriguingly, a lack of either *ARR16/17* or *CLE9/10* causes high cytokinin response in SLGCs, promoting and preserving SPCH expression, indicated by an increased SLGC division potential. Whereas, these mechanisms exert minimal effects on eventual stomatal patterning, it influences the way satellite stomata are generated. The negative feedback loop between SPCH target activation, cell type-specific negative regulation of cytokinin response factors, and transcriptional repression of SPCH driving ACDs in the stomata cell lineage may ensure the physiological adaptation of leaf growth and cell fate states to a given environmental setting. How other plant hormones participate in this complex molecular network is still unclear.

## Formation of ternary receptor complexes

Following signal perception at the plasma membrane, downstream intracellular components of RLK-mediated signaling are activated, typically through the formation of receptor complexes comprising a primary receptor and its co-receptor (Belkhadir et al., 2014). The near-universal co-receptor is BAK1/SERK3 (BRI1-ASSOCIATED RECEPTOR KINASE1/SOMATIC EBRYOGENESIS RECEPTOR KINASE3; Li et al., 2002; Nam and Li, 2002). The BAK/SERK family comprises five family members: SERK1–5 (Hecht et al., 2001). SERK1–4 regulate male gametophyte development, brassinosteroid (BR)-mediated growth, plant immunity, and cell-death control (Chinchilla et al., 2007; Heese et al., 2007; Li, 2010; Postel et al., 2010; Roux et al., 2011; Aan den Toorn et al., 2015). Intensive structural studies on several LRR–RLK–SERK complexes have unravelled how BAK/SERKs mediate receptor signaling through association with many different LRR–RLKs upon binding their respective ligands (Wang et al., 2008; Santiago et al., 2013; Sun et al., 2013a, 2013b; Wang et al., 2015; Hohmann et al., 2018).

Genetic evidence suggests that SERK members contribute redundantly to stomatal patterning (Meng et al., 2015). Higher-order *serk1 serk2 bak1 serk4* quadruple mutants display excessive stomatal clustering and developmental growth phenotypes reminiscent of the *er erl1 erl2* triple mutant, suggesting that these receptors may function together (Meng et al., 2015). Importantly, the stomatal cluster phenotypes of *bak/serk* higher-order mutants can be genetically uncoupled from BR signaling defects, indicating that BAK/SERKs enforces stomatal patterning together with ERf, not with BRI1 (Meng et al., 2015). Biochemical co-immunoprecipitation assays further demonstrate that SERKs form an EPF peptide ligand-dependent multi-protein receptor complex with both ER-TMM and ERL1-TMM with EPF2 and EPF1, respectively (Meng et al., 2015).

Interestingly, SERKs not only contribute to the EPF/EPFL peptide ligand dependent pathway but also to CLE9/10 peptide ligands and their receptor, HSL1. Upon receptor complex formation and peptide ligand binding, the binding affinity between CLE9/10 and HSL1 is higher in the presence of SERK family members (Qian et al., 2018). The unique ability of SERK family members to stabilize this specific receptor peptide ligand complex suggests that one primary function of this particular interaction might be to slow down the dissociation of peptide ligands from their respective receptors, a phenomenon that has been reported for other LRR–RLK–peptide ligand complexes upon co-receptor heterodimerization (Hohmann et al., 2018).

## Step by step—how downstream components integrate receptor-peptide signaling

Ligand-activated heterodimerization of primary LRR–RLKs and BAK/SERKs triggers phosphorylation and association of receptor-like cytoplasmic kinase (RLCK), which bridges the further downstream signal transduction (Liang and Zhou, 2018). Originally identified as a transducer of BR signaling, BRASSINOSTEROID SIGNALING KINASE (BSK) family RLCKs regulate immunity response with FLS2 (Kim and Wang, 2010; Shi et al., 2013; Nolan et al., 2020; Wang et al., 2020). A recent study provided genetic evidence supporting the concept that the BSK family might be the missing link between EPF-mediated receptor-activation and the MAPK cascade (Neu et al., 2019). A loss-of-function double mutant, *bsk1 bsk2*, results in a clustered stomata phenotype resembling that of *yda* or *er erl1 erl*2 triple mutants. BSK1 interacts with the kinase domain of the MAPKKK YDA (Neu et al., 2019). Thus, upon EPF-mediated receptor activation, a tertiary complex of ERf–TMM–SERK may physically interact with BSK1/BSK2 to transduce the phosphorylation-encoded extracellular information further on to YDA (Figure 1).

Formation of the EPFs–ERfs–TMM–SERKs (-BSKs) ligand–receptor signaling complex activates the downstream MAPK cascade, and this activation leads to the degradation of each bHLH TF module (Bergmann et al., 2004; Gray and Hetherington, 2004; Wang et al., 2007; Lampard et al., 2008, 2009; Horst et al., 2015). Consequently, while loss-of-function of MAPK-cascade components causes stomata overproduction, expression of constitutively active versions strongly inhibits stomatal development. Although it has been known for a while that the MAPK cascade plays a significant role in inhibition of SPCH, the molecular mechanism behind the phosphorylation-dependent degradation of each bHLH TF module individually remained elusive until now. A recent structure–function study demonstrated that activated MPK3/MPK6 associate first with SCRM through its bipartite motifs, triggering the subsequent phosphorylation and degradation of the SCRM–SPCH heterodimers, thus preventing entry into the stomata lineage (Putarjunan et al., 2019). Substitutions within the SCRM-KRAAM motif abolish the association with MPK3/6, resulting in stomatal overproduction, a phenomenon observed in *scrm-D* mutants (which possesses a KRAAM–to-KHAAM amino-acid substitution). Intriguingly, while the putative MAPK docking motif is highly conserved among vascular and nonvascular plants, the KRAAM motif can only be found in SCRM, SCRM2, and their orthologs. This has led to the hypothesis that, while MPK3/6 regulates myriads of developmental, environmental and immunity responses (Zhang, 2018), distinct binding motifs in MAPK substrates are used to cause specific developmental responses (Figure 1; Putarjunan et al., 2019).

In biological processes, kinases and phosphatases act as a phospho-switch to modulate and fine tune the activity of their respective substrates. So far there have been two phosphates identified, which further fine tune the signaling output in stomatal development. MAP KINASE PHOSPHATASE1 (MKP1) controls the phosphorylation status of MAPKs within stomatal precursors downstream of the MAPKKK YDA (Tamnanloo et al., 2018). *mpk1* mutant epidermis undergoes asymmetric entry division. However, Ms occasionally fail to differentiate, resulting in rose-petal like SLGC clusters, reminiscent of the *mute* mutant and *EPF1* overexpression phenotypes (Tamnanloo et al., 2018). *MUTE* expression is diminished in *mpk1* (Tamnanloo et al., 2018). These findings suggest that MPK1 counteracts with MPK3/6 in the EPF1–ERL1-mediated signaling pathway to promote M-to-GMC differentiation.

In addition to MKP1, a recent work revealed that subunits A1, A2, and A3 of the PP2A phosphatase promote stomatal development (Bian et al., 2020). Alteration in PP2A activity, either through higher-order *pp2a* mutants or through pharmacological impairment, suppresses stomatal production, indicative of a positive role of PP2A during stomatal development. Furthermore, PP2A-A subunits directly bind SPCH in vitro, suggesting that PP2A may function to regulate the phosphorylation-dependent equilibrium of SPCH protein. Future studies will hopefully illuminate how PP2A activity as well as its regulation is integrated within the ER–EPF2 receptor–peptide signaling module.

## How intrinsic polarity cues contribute to ACD and stomata pattern formation

**Figure 3:**
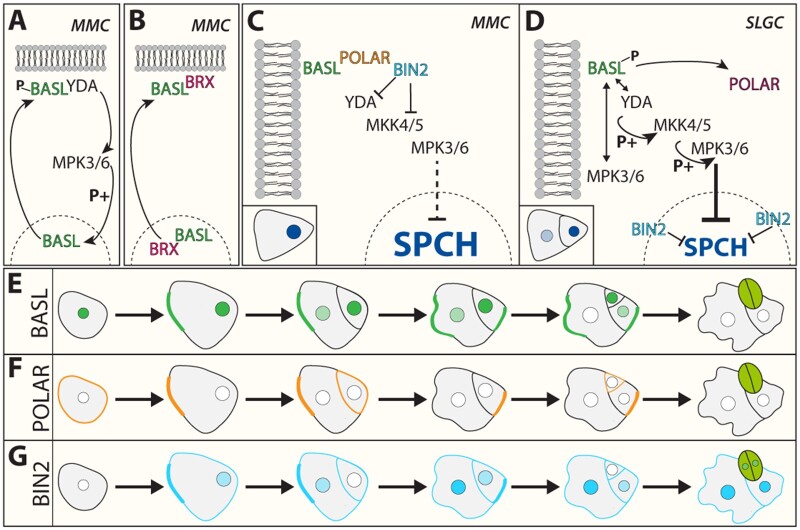
Intrinsic polarity cues contribute to stomatal development. The plant*-*specific polarity protein BASL (green) exhibits a dual localization pattern. While BASL accumulation can be detected in the nucleus during stomatal lineage progression, it additionally forms a polarized crescent in MMCs and meristemoids in the region distal to the future division plane and away from the migrated nucleus. This crescent localization is dependent on both MPK3/6 (A), as well as on members of the plant*-*specific BREVIS RADIX (BRX) (light red) family (B). Right before MMC asymmetric division (C), BASL and POLAR (orange) recruit the BIN2 (light blue) to the cellular cortex. This BASL-POLAR-BIN2 polarity module enables the inhibition of the YDA MAPK cascade via direct phosphorylation of YDA. This leads to an elevated accumulation of SPCH, hence promoting stomatal asymmetric division. To prevent further ACD in SLGCs, SPCH must be down regulated. To do so, BIN2 disassociates from POLAR after phosphorylating the latter and re-localizes into the nucleus to inhibit SPCH activity while POLAR is degraded (D). Furthermore, a positive feedback loop between BASL and the YODA MAPK cascade restricts further stomatal ACD in the SLGC through direct phosphorylation of SPCH. The specific localization pattern of the BASL-POLAR-BIN2 polarity module further fine tunes stomatal development (E–G).

Although receptor-mediated signal-transduction is critical for the regulation of stomatal development, evidence points towards a broader, more complex network of intrinsic polarity cues integrated with extrinsic signaling components that provide spatial information during cell division. ACDs give rise to cells of different sizes and shapes, potentially with different cell fates (Abrash and Bergmann, 2010). To achieve this, the mother cell has to determine an axis of polarity prior to mitosis in a way that the nucleus possesses an asymmetric position prior to division, orienting the division plane in relation to this axis (Rasmussen et al., 2011). In addition, during asymmetric amplifying divisions, SPCH activity must be strictly regulated in both Ms and SLGCs to sustain the stem-cell-like properties of the M. The plant specific polarity protein BASL promotes asymmetry via differentially regulating SPCH activity within two daughter cells, M and SLGC, within the stomatal lineage (Figure 3; Dong et al., 2009; Zhang et al., 2015, 2016). BASL displays a dynamic subcellular localization, accumulating first in the nucleus of pre-mitotic cells, followed by a highly polarized crescent in the region distal to the future division plane and away from the migrated nucleus (Dong et al., 2009). In loss-of-function *basl* mutants, a high proportion of symmetric cell divisions can be observed in MMCs. Furthermore, a domain analysis of BASL identified its C-terminal region as a prerequisite to direct polarized accumulation at the cell periphery, which was sufficient for polar cell growth (Dong et al., 2009). This suggests that the initial nuclear localization serves as a reservoir of BASL protein.

BASL polar localization and function is dependent on both MPK3/6, as well as on members of the plant-specific BREVIS RADIX (BRX) family (Figure 3, A and B; Zhang et al., 2015, 2016; Rowe et al., 2019). One model suggests that MPK3/6-dependent phosphorylation of BASL is essential to shift from its nuclear localization toward accumulating at the plasma membrane (Figure 3A; Zhang et al., 2015, 2016). Here, BASL directly associates with YDA, acting as a scaffold protein to polarly tether the MAPK complex to the cellular cortex of SLGCs. This provides a positive feedback loop between BASL and the YDA–MAPK cascade. Although the molecular details still remain unclear, this polar localization is necessary to inhibit SPCH activity within the SLGC. Conversely, it has recently been reported that members of the BRX family are also required for ACD within the stomatal lineage, forming a co-dependent equilibrium with BASL for both polarization and localization (Figure 3B; Rowe et al., 2019). BRXf and BASL physically interact to mutually influence localization, independent of the BASL-MAPK circuit, demonstrating the existence of a more complex core polarity system that enables the scaffolding, positioning, and segregation of additional proteins necessary for cell-fate commitment.

Polarity proteins from the POLAR LOCALIZATION DURING ASYMMETRIC DIVISION AND REDISTRIBUTION (POLAR) family function at the cortical site distal to the future division plane of premitotic MMCs and Ms in a BASL-dependent manner (Figure 3, C–G; Pillitteri et al., 2011). POLAR forms a scaffolding complex that controls the polarity of the GLYCOGEN SYNTHASE KINASE3/SHAGGY-like/BR-INSENSITIVE2 (BIN2) kinase. The scaffold complex enables the inhibition of the YDA MAPK cascade at the cell cortex before ACD via direct phosphorylation of YDA in a BIN2-dependent manner (Gudesblat et al., 2012; Khan et al., 2013; Houbaert et al., 2018). This, in turn, disrupts the balance of both nuclear and cytoplasmic MAPK signaling components, resulting in elevated accumulation of SPCH in the nucleus and promoting stomatal asymmetric division in Ms (Figure 3C). Soon after division, BIN2 phosphorylates POLAR at the BASL defined polarity site, resulting in the disassociation of BIN2 and its re-localization to the nucleus (Houbaert et al., 2018). The combined effort of nuclear-localized BIN2 activity, which directly phosphorylates SPCH and elevates MAPK signalling, further restricts ACD in the SLGC, resembling a fine-tuning mechanism of cell specification during leaf morphogenesis (Figure 3D; Gudesblat et al., 2012; Houbaert et al., 2018).

The intensive localization analyses of BASL-YDA-MPK3/6 as well as BASL-POLAR-BIN2 modules suggest that their ability to polarize and to form a complex in vivo emanates from BASL’s ability to self-organize in a polar manner upon phosphorylation by the YDA-MPK3/6 cascade (Zhang et al., 2015; Houbaert et al., 2018). This feedback circuit implies a mechanism in which a MAPK signaling gradient is formed descending from the BASL crescent toward the nucleus in a concentration-dependent manner (Shao and Dong, 2016). The idea of constitutive signaling gradients in the context of development has been identified in various biological systems. For instance, the small GTPase RAN, a key player in the “spindle self-organization” pathway in animals, maintains a gradient within a certain threshold at discrete positions around the chromosomes, controlling the activity of spindle assembly factors (Kalab et al., 2002; Clarke and Zhang, 2008; Zhang and Dawe, 2011).

With respect to a potential signal gradient during stomatal development, a recent publication identified new MAPK substrates, which regulate stomatal production in a positive manner (Xue et al., 2020). The authors reported that the MAPK SUBSTRATE IN THE STOMATA LINEAGE (MASS) 1, 2, and 3 function at the plasma membrane, negatively impacting the MAPK signaling cascade. Similar to BASL and in contrast to MASS3, both MASS1 and MASS2 initially localize to the nucleus and are re-localized toward the cell periphery upon MPK6 phosphorylation. However, overexpression of MASS2 results in clustered stomata and disregard of the “one-spacing rule” independently of BASL, since its localization remains unaffected (Xue et al., 2020). These data might reflect a potential relationship between the regulation of division reorientation by extrinsic signals triggered by ligand–receptor interaction and/or the YDA MAPK cascade through the control of a MAPK gradient within the stomatal lineage. With recent identifications of polarity proteins both in the evolutionarily conserved or broader versus tissue-specific contexts (e.g. Muroyama and Bergmann, 2019; Van Dop et al., 2020), our understanding of the mechanism underpinning intrinsic plant cell polarity is burgeoning. Whether shared extrinsic peptide signaling modules are co-opted to influence different polarity systems is an important future question.

## Variations in a theme: shared modules

**Figure 4:**
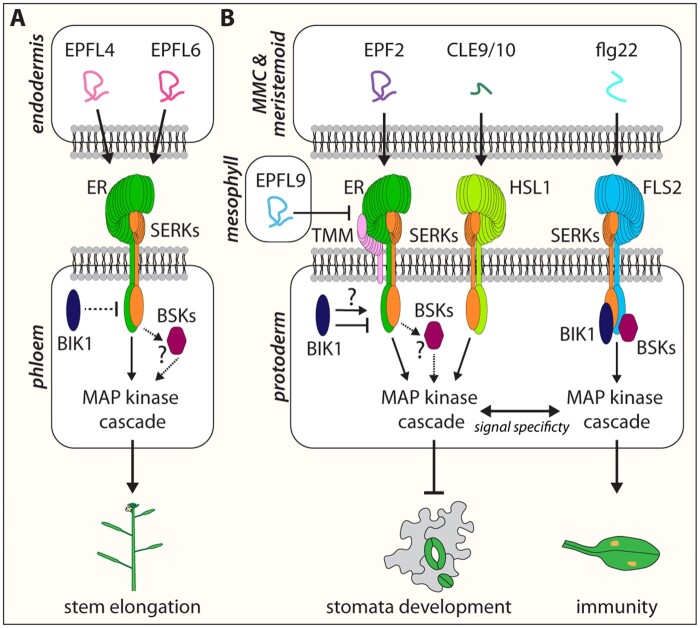
Signal specificity is maintained between different LRR–RLKs with shared intermediate components. ER mediates different developmental processes upon different peptide-ER-co-receptor formation. (A) During stem elongation EPFL4 and EPFL6 are secreted from endodermis cells into the apoplast to be perceived by ER in phloem cells in a noncell autonomous manner. Upon EPFL4/6-ER-SERKs formation, with the additional possibility of a higher order complex with BSKs (question mark) as well the RLCK BOTRYTIS-INDUCED KINASE1 (BIK1), a MAP kinase cascade is activated to transduce the extracellular information into the cell to further promote downstream effectors for stem elongation. Although stomata development depends also on the formation of an EPF/EPFL–ERf–SERKs complex, possibly with BSKs, to activate a MAPK cascade to overall inhibit stomata development (B), this peptide–receptor-co-receptor complex additionally requires the co-receptor TMM. Whether BIK1 also plays a role during stomata development is still unknown (question mark). Stomata development can be further fine-tuned through CLE9/10 peptides, which are perceived by the LRR receptor kinase HAESA-LIKE 1 (HSL1) and its coreceptor BAK1, enabling the activation of the same components of the MAPK cascade. It is unclear how the MAPK cascade is able to cope with the signal output of two different peptide–receptor signaling complexes for the same developmental process as well as create signal specificity between shared pathways, such as the flg22–FLS1–SERKs signaling module for plant innate immunity.

*ER* was originally described as a gene regulating inflorescence architecture, found as a spontaneous mutation in the commonly used accession Landsberg *erecta* (Rédei 1992; Torii et al., 1996). Through the analysis of stomatal development, upstream ligands, receptor complex modules, and intracellular phosphorylation cascades have been discovered. This raises a further question as to whether other aspects of ERf-mediated developmental processes use analogous, even shared signaling components. Indeed, two EPFL peptides, EPFL4 and EPFL6, promote inflorescence architecture as ligands for ER. Interestingly, EPFL4/6 are expressed in the stem endodermis, and phloem-expressed ER is sufficient to perceive these signaling peptides in a non-cell autonomous, paracrine manner (Figure 4; Uchida et al., 2012).

Like stomatal development, co-receptor BAK/SERKs and RLCKs BSK1 and BSK2 promote inflorescence architecture most likely with ER: higher-order *bak/serk* mutants as well as *bsk1 bsk2* double mutants develop shorter inflorescence with characteristic flower-bud clusters, a phenotype resembling that of *er* (Meng et al., 2012; Neu et al., 2019). Yet another RLCK, BOTRYTIS-INDUCED KINASE1 (BIK1), has been recently reported in the contexts of ER signaling. In contrast to BSK1/2, BIK1 plays a negative role in inflorescence development (Figure 4A; Chen et al., 2019). Loss-of-function *bik1* mutant plants develop significantly longer internodes and pedicles, as well as a looser inflorescence compared to wild-type plants. This phenotype could be partially rescued upon additional loss of *er*, indicating an antagonistic relationship between BIK1 and ER. Best known for its essential roles in FLS2 immune signaling and BR signaling, BIK1 acts downstream of different RLK modules to further fine tune or restrict the signaling output upon or before signal perception (Lozano-Durán and Zipfel, 2015). The EPFL4/6-ER-BAK/SERK-BSK1/2-BIK1 peptide-receptor module is most likely transduced via a MAPK cascade, again involving MKK4/5 and MPK3/6 (Figure 4A; Meng et al., 2012). Loss-of-function in YDA MAPKKK exhibits severe, pleiotropic growth defects (Lukowitz et al., 2004), which somewhat resemble the *er erl1 erl2* triple mutant (Shpak et al., 2004). However, their genetic interactions are complex (Bemis et al., 2013).

Altogether, these findings highlight that EPF/EPFL-ERf-mediated signaling pathways in stomatal development and inflorescence growth are variations on a theme—pathways composed of the same/paralogous modules (Figure 4). Although the downstream components of such ERf-mediated developmental processes often remain unknown, these variations on a theme become even more visible when other EPF/EPFL-ERf-mediated signaling pathways are taken into consideration. During leaf margin morphogenesis, for instance, auxin responses are maintained at tips of the teeth to promote their growth (Kawamura et al., 2010; Heisler and Byrne, 2020). This auxin response represents a culmination of a feedback circuit between the EPFL2/ERf peptide–receptor module to restrict and define auxin maxima (Tameshige et al., 2016). Auxin shapes the site of EPFL2-ERf signaling at the boundary of each leaf teeth primordium by repressing *EPFL2* expression from the leaf teeth tips while promoting *ERL2* expression at the leaf teeth tips. The EPFL2-ERf signaling in turn restricts the auxin maxima in the tip to enable directed leaf teeth growth.

In addition to its role during leaf margin morphogenesis, a recent study found that differential expression of EPFL2 and EPFL9/STOMAGEN, resulting in a reginal activation of specific ERf members, couples ovule initiation with fruit growth (Kawamoto et al., 2020). While EPFL2 acts predominantly through ERL1 and ERL2 to control the initiation and spacing of ovule primordia during gynoecium and fruit growth, EPFL9/STOMAGEN, acting from the carpel wall, mainly promotes fruit growth through ER. Intriguingly, EPFL9/STOMAGEN fail to compensate here for the loss of *epfl2* when expressed under the *EPFL2* promoter, indicating that EPFL9/STOMAGEN can antagonize EPFL2 functions during fruit growth, a phenomenon best described in stomatal development.

All in all, EPF/EPFL-ERf-mediated signaling pathways and their same/paralogous modules became indispensable for various aspects of plant growth and development. Many signaling components, such as BAK/SERKs, RLCKs, and MAPK cascades, are even shared with broader signaling pathways in development, environmental response, and immunity (Kim and Wang, 2010; Shi et al., 2013; Nolan et al., 2020; Wang et al., 2020). This highlights two important questions of signal discrimination and specificity. First, how can ERfs discriminate different EPF/EPFL peptides and properly ensure specific outcomes? Second, how can different signaling pathways, such as those of stomatal development and immunity, maintain specificity?

One critical factor distinguishing EPF/EPFL-ERf-mediated stomatal development from inflorescence growth is TMM (Figure 4). TMM is specifically expressed in the epidermal layer, and whereas EPF1 and EPF2 require TMM to activate ERf members, EPFL6 (also known as CHALLAH; CHAL) and EPFL4 (CHAL-LIKE2) do not (Abrash and Bergmann, 2010; Abrash et al., 2011). In the absence of TMM, ERf in epidermal cells can be activated by EPFL4/6 which bleed-through from endodermis to epidermis and inadvertently suppress stomatal development in stems and hypocotyls (Abrash and Bergmann, 2010; Abrash et al., 2011). Consistently, *epfl4/5/6* triple mutation reverses the stomata-less phenotype of *tmm* stems and hypocotyls (Abrash et al., 2011). More recent structural biology and cell biology studies support this model: EPFL4/6 binds with 10 time higher affinity to ERL1 than to the ERL1–TMM complex (Lin et al., 2017). Likewise, ERL1 can perceive EPFL6 and rapidly undergoes endocytosis in the absence of TMM (Qi et al., 2020).

Understanding how signal specificity is maintained between different LRR–RLKs with shared intermediate components, such as stomatal development and immunity signaling, is an important outstanding question. Evidence has been provided that both the EPF–ERf and flg22–FLS2 immunity pathways exhibit antagonistic interactions at the MAPK cascade (Figure 4B;Sun et al., 2018). On the other hand, a more recent, newly designed genetic tool that can constitutively activate BAK/SERK-dependent LRR–RLKs suggests the maintenance of basal signal specificity between ERf and FLS2 pathways when they are expressed within the context of stomatal cell lineages (Hohmann et al., 2020). Harnessing new tools and approaches will be required to further delineate the molecular mechanisms preventing signal interference.

## Conclusion and perspectives

Significant progress has been made in the past two decades to unravel the molecular mechanisms behind stomata development. The discovery of specific receptor–peptide signaling complexes, transducing their extracellular perceived peptide signals onto a MAPK cascade to further inhibit specific TFs, has dramatically enhanced our understanding of cell state transition and differentiation within the stomatal lineage. The identification of intrinsic polarity cues, contributing to ACD, has provided us a unique opportunity to understand division plane switch and the mechanisms behind protein polarization at the plasma membrane in plants. There are still very big, open questions to be addressed (see also Outstanding Questions Box). For example, what molecular mechanisms connect external signaling factors with the intrinsic polarity cues? And how, through the evolution of land plants, have different plant species co-opted and re-wired these signaling and polarity modules to achieve specific stomatal patterns optimized to their ecological niche? Recent studies in monocots, including rice (*Oryza sativa*), grass species (*Brachypodium dystachyon*), and moss (*Physcomitrella patens*), reveal new exciting insights (Caine et al., 2016; Chater et al., 2016; Raissig et al., 2017; Abrash et al., 2018; Hepworth et al., 2018; Lu et al., 2019; Wu et al., 2019).

The ability of plants to regulate themselves to the atmospheric balance and water cycles of our planet is dependent on the precise control of stomatal development and distribution. Since key regulators of stomata development are conserved in grass species and most land plants (see also Endo and Torii, 2019), deciphering the molecular network underlying these processes could well uncover a promising trait for agricultural application. It is therefore crucial to continue basic research to fully understand the molecular mechanisms behind these developmental processes and how plants integrate external signals to optimize stomatal formation. Applying new genetic tools, we may be able to manipulate stomatal development and patterning in our favor to increase agricultural harvests and reinforce plants to an ever-changing climate.

### Accession numbers

Please see Table 1: Regulators of Stomatal Development for an overview of all accession numbers of all major genes and proteins mentioned in this review.

## Notes

All authors conceived, drafted, edited, and critically reviewed the text and figures of this article.
